# Association Between *Helicobacter Pylori* Infection and Primary Open Angle Glaucoma

**DOI:** 10.5539/gjhs.v6n7p13

**Published:** 2014-09-18

**Authors:** V. Samarai, N. Sharifi, Sh. Nateghi

**Affiliations:** 1 Department of Ophthalmology, Imam Hospital, Urmia University of Medical Sciences, Urmia, Iran; 2 Urmia University of Medical Sciences, Urmia, Iran

**Keywords:** *Helicobacter Pylori*, primary open angle glaucoma, Eliza

## Abstract

**Aim:**

To compare the prevalence of Pylori infection in patients with primary open angle glaucoma (POAG) and control group with cataract.

**Methods:**

This is a prospective case-control study. The participants were organized in two groups. First group (case) consisted of 35 patients with POAG and second group consisted of 35 age matched participants with cataract whose optic disk could be evaluated. Serum levels of anti H. pylori IgG antibody were evaluated with the method of ELISA.

**Results:**

The seroprevalence of Pylori infection was 89.1 % (33 of 37) in patients with POAG and 59.5 % (25 of 42) in the control group. The difference was significant (P=0.008). The odds ratio for association between Pylori and POAG was 5.69 and the range of 95% confidence interval was from 1.58 to 20.50.

**Conclusion:**

This study suggests that *Helicobacter Pylori* infection might be associated with **primary open angle glaucoma.**

## 1. Introduction

Primary open-angle glaucoma (POAG) is one of the leading causes of blindness in the world, with prevalence of 70 million patients worldwide (Kountouras et al., 2003; E. C. Kim, Park, & M. S. Kim, 2010). It is a chronic optic neuropathy characterized by atrophy and increased cupping of optic disk. Many aspects of its pathogenesis remain unknown but some significant risk factors are advanced age, African origin, familial history of glaucoma, and elevated intraocular pressure (Cantor, Fechtner, & Michael, 2004, 2005; Bron et al., 2008). According to evidences, other probable factors involved in POAG include: changes in endothelin-dependent vascular regulation (Orgul, Prunte, & Flammer, 1998; Sugiyama et al., 1995; Luscher et al., 1995; Tezel et al., 1997; Gass et al., 1997), cytokine-dependent platelet aggregation causing impaired ocular blood flow (Haefliger et al., 1999; et al., 1992; Wakefield & Lioyd, 1992), influencing apoptotic process and apoptotic loss of specific populations of optic disk neurons (Kountouras et al., 2002), and other autoimmune mechanisms which may induce and/or exacerbate glaucomatous optic neuropathy (Cartwright et al., 1992; Romano et al., 1995; Tezel, Edward, & Wax, 1999). since the discovery of *H. pylori* in 1982, there has been an explosion of data about its role in gastric ulcer (Cohen, 2000) and gastric carcinoma and its association with some other diseases like cerebrovascular disorders, vascular disorders, coronary heart disease and some autoimmune conditions such as Sjogren’s syndrome and immune thrombocytopenicpurpura (Kountouras et al., 2003; Hong et al., 2007). Another probable association is glaucoma which some of Pathophysiological mechanisms thought to link the *H. pylori* include: 1- promoting platelet and platelet-leucocyte aggregation, 2- releasing pro-inflammatory and vasoactive substances, 3- causing the development of cross mimicry between endothelial and *H. pylori* antigens, 4- influencing apoptotic process (Kountouras, 2008). Recent evidences show the controversies about association between *H. pylori* and glaucoma (Kountouras, 2004). For instance studies in Greece, china, Iran, and Australia have reported considerably higher prevalence of *H. pylori* infection in patients with open angle glaucoma than in patients without it (Hong et al., 2007, Kountouras et al., 2001; Abrishami et al., 2007; Kountouras et al., 2008; Galloway et al., 2003). However other studies in Canada and Iran have not reported statistically significant differences between them (Kurtz et al., 2008; Abdollahi et al., 2005; Izzotti et al., 2009). Considering these controversies this study was designed to compare the prevalence of *H. pylori* infection in Iranian patients having POAG and control group of participants with cataract.

## 2. Methods

This is a case-control study performed at Eye Center of Imam Hospital, Urmia Medical Science University, Iran. Approval was obtained from the ethics committee of university.

### 2.1 Patients

First group (cases) included 35 consecutive patients diagnosed as having POAG. Inclusion criteria were:

- Intraocular pressure (IOP) ≥21 mmHg.
- Open angle of anterior chamber in gonioscopy.
- Glaucomatous optic nerve head changes, including rim thinning, notching in the inferior or superior temporal area of the optic nerve head, or total glaucomatous cupping.
- Visual field changes such as generalized depression, paracentral scotoma, nasal step.

Patients with the history of angle closure glaucoma or other kinds of glaucoma were excluded. Complete ocular examinations of patients including applanation tonometry (by calibrated Goldmann tonometer) and gonioscopy (by Goldmann3-mirror goniolens). The optic disk was further evaluated with +78 D lens, and the visual field was assessed by Humphrey’s automated perimeter using the SITA Standard program. Control group was selected from the ophthalmology clinic at the same hospital. This group consisted of 35 consecutive age and sex-matched participants with cataract whose optic disk could be evaluated. Control participants underwent slit-lamp examination, indirect ophthalmoscopy, IOP measurement, and visual field examination. None of them had glaucomatous optic nerve head changes or visual field changes and their IOP was less than 21 mmHg. Exclusion criteria for both groups included diabetes mellitus, upper GI diseases, severe systemic diseases or neoplasms, myopic refractive error exceeding -10 dioptre, and serious eye diseases except glaucoma and cataract. Furthermore, participants were excluded if they had received drugs such as H2-receptor antagonists, proton pump inhibitors, antibiotics, bismuth compounds, or non-steroidal anti-inflammatory drugs in the previous 4 weeks.

### 2.2 Serologic Assays

Informed consent was obtained from all participants. In order to determine the serum levels of anti-Pylori IGg antibody, venous blood samples were collected and centrifuged at 3000r.p.m. for 10 min to obtain serum, and then were stored at -20 ^o^C (20-25 days). All samples were evaluated with ELISA method (Pishtaz teb kit) and by the certain laboratory. Considering the cut off standard of the kit recommended by manufacture, the standard higher than 10 was proposed as seropositive (sensitivity and specificity more than 98%).

### 2.3 Serologic Assays

All analyses were performed with SPSS software (version 11). Using T-test and Chi-square test. P-value less than 0.05 were considered significant. Considering type I error (α):5% and type II error (β):5%, the sample size of 35 patients was estimated for each group. Odds ratio was achieved (Majazi-Dalfard et al., 2013).

## 3. Results

The demographic and clinical characteristics of participants are summarized in Table 1 and Table 2:

**Table 1:** Clinical characteristics of participants

Characteristic	Patients: 37	Controls: 42
Age (years)		
Mean	73.05 ± 7.3	67.21 ± 9.5
Range	58 – 84	40 – 81
Sex		
Male/Female	20/17	18/24

**Table 2:** Serology test results

Characteristic	Patients: 37	Controls: 42
Seropositive for *H. pylori*	33 (89.1 %)	25 (59.5 %)
Age (year)		
Mean	72.94 ± 7.5	66.29 ± 10.5
Sex		
Male / Female	18/15	12/13

There is no significant difference in age and sex ratio between study groups. The seroprevalence of *H. pylori* infection was 89.1 % in patients with POAG and 59.5 % in the control group. The difference was significant (P=0.008). The odds ratio for association between *H. pylori* infection and POAG was 5.69 and the range of 95% confidence interval was (1.58–20.50).

## 4. Discussion

According to this study there is a probable association between *H. pylori* infection and Primary Open Angle Glaucoma. Comparing serology results reveals significantly higher prevalence of *H. pylori* infection in patients with POAG. By consecutive sample collecting of patients and control subjects authors tried to reduce possible selection bias. Serologic detection of Anti *H. pylori* IgG is the gold standard for diagnosis of *H. pylori* infection. Mucosal biopsy is an invasive method and non-invasive tests are more preferable for screening (Kountouras et al., 2003). In this study serologic evidence of *H. pylori* infection was found in 89.1% of patients with open angle glaucoma (POAG) and 59.5% of patients with cataract (as the age and sex matched control s) The difference was statistically significant (P=0.008, OR=5.69). Long term record of this association belongs to Greece. The primary study; using several methods such as histologic analysis and rapid urease slide test (CLO test) for biopsy and saliva samples and also serologic tests, reported more frequency of *H. pylori* in glaucoma patients than anemic control group (p<.001) (Kountouras et al., 2001). The following study reported that elimination of *H. pylori* might have effectively improve glaucoma parameters (p<.001 for intraocular pressure; p≤.01 for visual field), giving the idea of possible casual relation between *H. pylori* and glaucoma (Kountouras et al., 2002). The next article of these series supported the theory of induction of apoptosis with *H. pylori* infection (Kountouras, 2004). Another study reported a probable genetic susceptibility for both *H. pylori* and POAG, suggested this infection as an environmental genetic cloning risk factor for POAG (Kountouras et al., 2004). Another study in china performing C-urea breath method reported significantly higher prevalence of *H. pylori* in patients with POAG (54.2%) than in control group (20.8%) (p=0.017 OR=4.49 CI 95%=1.26-16.01) (Hong et al., 2007).

Some studies reported conflicting results. For instance, in one study seropositivity of *H. pylori* was detected in 60.8% of glaucoma patients compared with 61.1% control patients having cataract. Also serum levels of Cag-A product was measured in both groups. It was reported that either *H. pylori* infection or seropositivity for virulent Cag-A bearing *H. pylori* strains do not have significant association with any type of glaucoma (P=0.88 for *H. pylori* and P=0.67 for Cag-A) (Kurtz et al., 2008). Other disagreeing studies about the relation between *H. pylori* infection and POAG carried out in Canada (Galloway et al., 2003) and Iran (Abdollahi et al., 2005). Some mechanisms probably involved with effect of *H. pylori* infection in pathophysiology of POAG include:

- Releasing vasoactive and pro-inflammatory factors, like eicosanoids (prostaglandins, leukotrienes), cytokines (interleukins 1, 6, 8, 10, 12, interferon ϒ, tumor necrosis factor [TNF]α), and acute phase proteins (C-reactive protein, fibrinogen) (Kountouras, 2004), and particularly endothelin1, inducing vasoconstriction of anterior optic nerve vessels and nitric oxide, modifying tonicity of ophthalmic artery, could cause glaucomatous changes in globe.
- Stimulating platelet and platelet-leukocyte aggregation leading to impaired ocular circulation.
- Developing a cross mimicry between endothelial and *H. pylori* antigens.
- Promoting mononuclear cells to induce a tissue factor-like procoagulant activity which turns the fibrinogen into fibrin (Kountouras et al., 2004).
- Presence of a common genetic factor that makes patients with glaucoma more susceptible to infectious diseases like *H. pylori* (Hong et al., 2007).
- Causing apoptosis activity by sharing FAS/FASL and mitochondrial-mediated apoptotic pathways between *H. pylori* and glaucoma (Kountouras, Zavos, & Chatzopoulos, 2004).

The most probable mechanism is the autoimmune reaction which anti-*H. pylori* antibodies in circulation might get through the blood-aqueous humor barrier, condensed in aqueous humor and induce or aggravate glaucomatous damages (Kountouras, 2003). One of limitations of our study was the small sample size achieved by following formula:


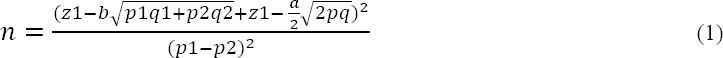


This was based on high difference in seropositivity among case and control group in a study with relatively similar samples in Iran (18). Using serologic analysis is another limitation of our study. Ethnical and financial issues not allowed us to performe invasive methods like endoscopy and biopsyor UBT. There is no significant difference in age between case and control group, so age probably is not confounding factor in this study.

## 5. Conclusion

This study is using serologic analysis of anti *H. pylori* antibody, could find a significant relationship between *H. pylori* infection and primary open angle glaucoma. We suggest 
